# The giant titin: how to evaluate its role in cardiomyopathies

**DOI:** 10.1007/s10974-019-09518-w

**Published:** 2019-05-30

**Authors:** Amar Azad, Giulia Poloni, Naeramit Sontayananon, He Jiang, Katja Gehmlich

**Affiliations:** 1 Division of Cardiovascular Medicine, Radcliffe Department of Medicine and British Heart Foundation Centre of Research Excellence, University of Oxford, Oxford, OX3 9DU UK; 2 Swansea University Medical School, Swansea, SA2 8PP UK; 3 Institute of Cardiovascular Sciences, University of Birmingham, Birmingham, B15 2TT UK

**Keywords:** Titin, Animal models, Induced pluripotent stem cell derived cardiomyocytes, CRISPR/Cas9, Genome-engineering, Cardiomyopathy

## Abstract

Titin, the largest protein known, has attracted a lot of interest in the cardiovascular field in recent years, since the discovery that truncating variants in titin are commonly found in patients with dilated cardiomyopathy. This review will discuss the contribution of variants in titin to inherited cardiac conditions (cardiomyopathies) and how model systems, such as animals and cellular systems, can help to provide insights into underlying disease mechanisms. It will also give an outlook onto exciting technological developments, such as in the field of CRISPR, which may facilitate future research on titin variants and their contributions to cardiomyopathies.

## Introduction

**Fig. 1:**
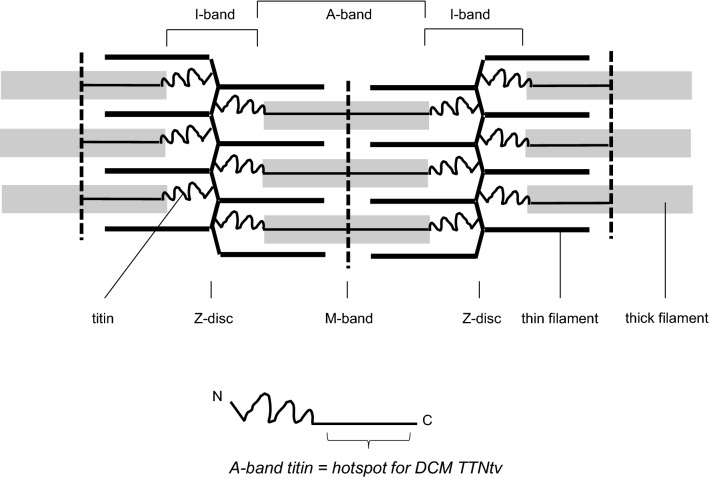
Schematic drawing of the sarcomere. Each titin molecule (black) spans half the sarcomere from the Z-disc to the M-band, linking thin filaments (bold black) and thick filaments (grey). The positions of I-band and A-band are also indicated. Bottom: The amino-terminus (N) and the carboxy-terminus (C) of an individual titin molecule are marked (bottom of figure). The A-band portion of titin as a hotspot for DCM-causing variants is indicated. For more detailed schemes of titin domain structure please see Bang et al. (2001), Lange et al. (2006)

Titin, historically also known as connectin (Maruyama 1976), is the largest protein known: with 3.4–3.9 megadalton it is 100 times larger than an “ordinary protein”, e.g. the house-keeping enzyme Glyceraldehyde 3-phosphate dehydrogenase, GAPDH. It is specifically expressed in striated muscle and has important structural and signalling functions for the contractile units, sarcomeres. Each titin molecule spans half a sarcomere from the Z-disc to the M-band (Fig. 1). Decades of research have shed light on its functions, structure, binding partners and post-translational modifications, for reviews see e.g. Koser et al. (2019), Kruger and Linke (2011), Lange et al. (2006), Zacharchenko et al. (2015). In this review, we will discuss the genetics of titin with respect to cardiac disease and how vertebrate animal and cellular models provide insight into the patho-mechanisms related to titin variants. The equally important topic of the role of titin in skeletal muscle disease has been discussed elsewhere (Ottenheijm and Granzier 2010; Savarese et al. 2016).

## Identification of cardiomyopathy-associated variants in titin

The enormous size of the human titin gene (*TTN*, 363 exons) makes it difficult, expensive and time-consuming to interrogate by conventional mutation screening approaches such as Sanger sequencing. Additional complicating factors include (i) alternative splicing, generating various differentially expressed isoforms in tissues and (ii) that a large portion of *TTN* consists of repetitive tandem Immunoglobulin-like encoding sequences and that variants in these regions may have very little effect on *TTN* length and function (Greaser 2009).

In 1999, the *TTN* locus (2q31) was associated with familial autosomal dominant dilated cardiomyopathy (DCM) for the first time (Siu et al. 1999). In the same year, Satoh and colleagues reported the first *TTN* single-base-pair missense variant in hypertrophic cardiomyopathy (HCM) (Satoh et al. 1999).

Advancement of next-generation sequencing technologies in the last decades has made genetic analyses of *TTN* more accessible: it can now be interrogated as part of screening for the whole genome, the whole exome or targeted panel of genes in a rapid and cost-effective manner (Norton et al. 2012). In a landmark study in 2012, Herman et al. conducted a genetic analysis of a large number of DCM, HCM and control cases and reported that *TTN* truncating variants (TTNtv, i.e. variants leading to a premature stop codon in *TTN*) are a common cause of DCM, occurring in approximately 25% of familial and 18% of sporadic cases, but only present in 1% of HCM cases. Spatial distribution of variants found in HCM subjects and controls was distinct from that of DCM subjects. The DCM variants were predominantly located in the A-band region. This data has been integrated with RNA sequencing (RNAseq) data from diseased left ventricular samples to help understanding the role of TTNtv (Roberts et al. 2015). This study also determined the mean usage of each *TTN* exon, denoted as the proportion of transcripts that incorporate each exon (percentage spliced in, PSI) and they estimated that 50% of the TTNtv identified in healthy controls occur in low PSI exons. Therefore, variants located in high PSI exons, including both N2BA and N2B isoforms, are strongly enriched in DCM patients versus controls. The penetrance of TTNtv in DCM appears to be strongly correlated with its position; distal I-band and all A-band variants show larger odd ratios compared to very proximal or distal ones (Schafer et al. 2017).

TTNtv may cause the generation of short truncated *TTN* isoforms unable to span between the M-line and the Z-disc, or normal-sized *TTN* isoforms that attach poorly to the Z-disc (Neiva-Sousa et al. 2015). In either case, the presence of abnormal *TTN* isoforms in the cardiomyocytes results in poorly contractile sarcomeres or may lead to DCM onset.

TTNtv have also be identified in the general population. From analyses of over 4500 control subjects TTNtv have been identified in 1.6% in individuals of African descent and 1.5% in individuals of European descent (Roberts et al. 2015). Two different independent studies estimated that 3% of the population carries a TTNtv allele (Golbus et al. 2012; Herman et al. 2012). In a recent study, a similar frequency of TTNtv (~ 2%) was estimated in a cohort of Chinese HCM patients and one of healthy Chinese controls (Zhang et al. 2017). This study showed for the first time that TTNtv increased the risk of cardiovascular death in patients with HCM and therefore they assigned a role to TTNtv in the phenotypic expression of the disease.

While the interpretation of TTNtv remains difficult due to the high frequency of these variants across normal populations and the lack of penetrance, the situation is far more complex for *TTN* missense variants, which have been identified in patients affected with different cardiac conditions, such as DCM, HCM, arrhythmogenic cardiomyopathy, restrictive cardiomyopathy and left ventricular non-compaction cardiomyopathy (Arimura et al. 2009; Gerull et al. 2002; Hastings et al. 2016; Itoh-Satoh et al. 2002; Matsumoto et al. 2005; Peled et al. 2014; Satoh et al. 1999; Taylor et al. 2011). However, it is likely that most of them act as ‘benign’ variants and do not have a role in the development of disease (Begay et al. 2015). In support, a comprehensive study of 530 DCM patients found no enrichment of missense or in-frame small insertions/deletions in *TTN* when compared to large population cohorts (Akinrinade et al. 2019). Nevertheless, for a rare subset of *TTN* missense variants, there is clear evidence for their role in the pathogenesis of cardiomyopathies, based on co-segregation studies in large families and in vivo disease models, e.g. Gerull et al. (2002), Hastings et al. (2016).

Although *TTN* plays a major role in the onset of DCM, due to its complexity, it is currently excluded from the list of genes to be reported as secondary findings by the American College of Medical Genetics (Kalia et al. 2017). When identified in patients, variants in *TTN*, especially missense ones, are often ignored in the first instance. To distinguish the rare disease-relevant variants from the majority of benign ones is a major, still unresolved challenge in clinical practice. Databases and bioinformatics tools are developed to help with the evaluation and interpretation of *TTN* variants (Table 1) and novel cellular model systems (see below) may help to address this issue experimentally in the future, as it has already been demonstrated for other cardiomyopathy genes (Ma et al. 2018).

**Table 1:** Online resources to evaluate titin variants

Name	Link	References
Titin variants in dilated cardiomyopathy	https://www.cardiodb.org/titin/	Roberts et al. (2015)
Leiden open variation database	https://databases.lovd.nl/shared/genes/TTN	Fokkema et al. (2005)
Genome aggregation database	https://gnomad.broadinstitute.org/gene/ENSG00000155657	Karczewski et al. (2019)
Titindb	http://fraternalilab.kcl.ac.uk/TITINdb/	Laddach et al. (2017)

## Insights into titin-related cardiomyopathy studying human hearts

Given the scarcity of human cardiac tissue for research purposes, especially from healthy control individuals, it is not surprising that only few studies have looked at molecular changes in the hearts of cardiomyopathy patients carrying titin variants (Roberts et al. 2015; Vikhorev et al. 2017). The main outcome of these studies is that no truncated titin proteins could be detected in individuals with TTNtv and that overall levels of titin transcript and protein are normal. Likewise, the isoform ratio N2B/N2BA is not altered. These findings argue against a haplo-insufficiency mode of action, yet support a dominant negative mode of action, but not through a poisonous-peptide mechanism. RNAseq data from 34 DCM patients with TTNtv identified alterations in mitochondrial function and fibrosis as molecular hallmarks of the disease (Verdonschot et al. 2018); however, these are unspecific features of many cardiomyopathies (Schirone et al. 2017).

## Modelling titin variants in animal models

Whole organism models are valuable tools to study the consequences of titin variants on the hearts. Important insights on titin function were gained from studying shorter titin-like proteins in invertebrates, such as twitchin in *C. elegans* and kettin in Drosophila; for reviews see Benian et al. (1999), Bullard et al. (2005), Ferrara et al. (2005). Zebrafish and mouse are the most commonly used animals to study cardiac functions of titin, but rat models and even a dog model also exist.

### Zebrafish models

Traditionally, zebrafish is a popular model organisms because of the ease of genetic modulation via random mutagenesis or morpholino oligomers, its short reproductive span and the transparent heart of its embryos, facilitating in vivo imaging (North and Zon 2003).

The *pickwick* mutant line is characterised by reduced contractility of its heart and poor myofibrillar maturation without mature sarcomeres (Xu et al. 2002), it is embryonic lethal. A homozygous single base pair change in the N2B region of titin, introducing a stop codon, was found to be the underlying genetic cause. It soon became apparent that there are two titin orthologs in zebrafish (*ttna* and *ttnb*) and that both the N2B-exon and N2A-exon containing isoforms of *ttna* are required for sarcomere assembly in the heart, while *ttnb* is dispensable for cardiac contractility (Seeley et al. 2007). An internal promotor, giving rise to the *Cronos* transcript, a C-terminal titin isoform, may explain why the position of truncation modulates disease severity, as was shown for skeletal muscle (Zou et al. 2015). However, there is still controversy about the evolutionary conversation and relevance of the *Cronos* transcript (Deo 2016; Shih et al. 2016).

Moreover, the zebrafish model has provided insight into the increased risk of atrial fibrillation in individuals carrying TTNtv (Ahlberg et al. 2018). A heterozygous TTNtv resulted in sarcomere defects in both ventricular and atrial chamber in the adult fish, as well as fibrosis and increased PR interval. These pathological changes may form a substrate for atrial fibrillation.

### Rodent and large animal models

Mice are by far the most established model organism to study gene function and its perturbation through genetic variants. As the global knock-out of titin is embryonic lethal (Radke et al. 2019), studies deleted important regions of the protein, such as the M-band portion (Gotthardt et al. 2003; Radke et al. 2019), the N2B region (Radke et al. 2007) and the PEVK region (Granzier et al. 2009), to understand the contributions of these regions to titin’s functions. All manipulations lead to disturbances in cardiac function, however the exact phenotype (DCM, hypertrophy or atrophy) depends on the deleted region.

Gramlich et al. (2009) introduced the human DCM-causing TTNtv c. 43628insAT (Gerull et al. 2002) into a mouse model. In the homozygous setting the variant is embryonic lethal, while heterozygous mice have no overt phenotype. However, upon chronic challenge with angiotensin or upon pressure overload via trans-aortic constriction for 2 weeks, the heterozygous mice developed features of DCM, namely systolic dysfunction and dilatation, while wild-type mice undergoing the same treatment had preserved cardiac dimensions and function. Also, TTNtv mice had increased levels of fibrosis upon both challenges (Gramlich et al. 2009; Zhou et al. 2015). The angiotensin-induced phenotype of the mouse model could be rescued by antisense-mediated exon skipping, removing the premature stop from the transcript (Gramlich et al. 2015). Another successful therapeutic approach to improve cardiac function in these mice was inhibition of miR-208b, a micro-RNA shown to be upregulated in the model (Zhou et al. 2017).

Rat models have been generated carrying TTNtv in either the Z-disc or the A-band portion of titin (Schafer et al. 2017). Like their mouse counterparts, homozygous rats were not viable. Young heterozygous rats (< 8 months) have preserved cardiac function, but showed signs of concentric remodelling. In old rats (> 1 year), mild systolic dysfunction, not quite reaching statistical significance in all parameters, was observed, irrespective of the position of the variant. The main value of the models is that ribo-sequencing and RNAseq data revealed that *TTN* transcripts with an A-band TTNtv were transcribed and translated as far as the premature nonsense codon, where the translation stops; whereas the premature stop codon of the TTNtv in the Z-disc did not prevent translation downstream. Regardless the position of the variant within the protein, TTNtv lead to nonsense-mediated degradation of the mutant allele and a signature of perturbed cardiac metabolism but they result in different translational footprints (Schafer et al. 2017).

A further study subjected the rat model with the TTNtv at the Z-disc to pressure overload by trans-aortic constriction. This resulted in mild diastolic dysfunction, increased fibrosis and reduced capillary density. Additionally, more apoptosis was observed in these mice (Ye et al. 2018). Finally, a more detailed molecular analysis of both rat models revealed impaired autophagy and mitochondrial defects leading to reactive oxygen species production and increased acetylation of mitochondrial proteins as disease mechanisms (Zhou et al. 2019). The study further demonstrated that mTOR inhibitor rapamycin can rescue the autophagy defects in these rat models, leading to the speculation that this could be an intervention to ameliorate ventricular remodelling associated with TTNtv.

Furthermore, a dog model of titin missense variant exists (Meurs et al. 2019), this homozygous or heterozygous missense change in immunoglobulin domain I71 in the N2BA isoform was identified in a Doberman pinscher dog pedigree with familial DCM and a high incidence of sudden cardiac death.

## Modelling titin variants in induced pluripotent stem cell derived cardiomyocytes

**Fig. 2:**
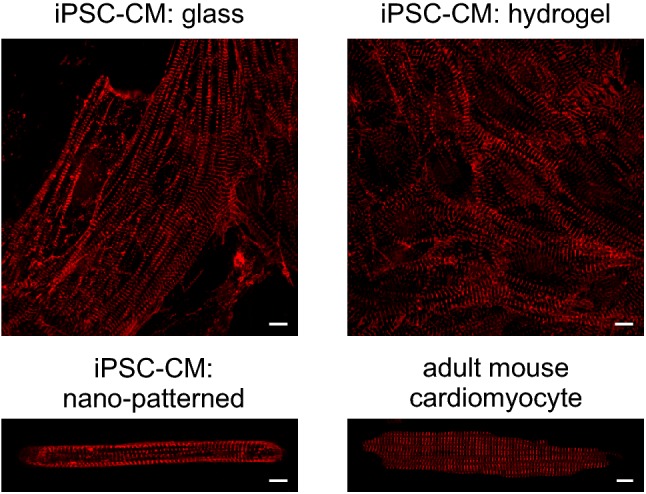
Immature phenotype of iPSC-CM in conventional 2D cultures: Normal Kolf2 iPSC (Streeter et al. 2017) were differentiated into iPSC-CMs using standard methods (Burridge et al. 2015) and plated on day 20 on different substrates: matrigel coated glass (top left), matrigel coated hydrogel (top right, 7 kPa) and nano-patterned matrigel coated hydrogel (bottom left, see Ribeiro et al. (2015)). 30 days after induction of differentiation, cells were fixed and sarcomeric structures visualised with an antibody direct against alpha-actinin (EA53, Sigma). For comparison, an isolated mouse cardiomyocyte stained with the same antibody is shown (bottom right). Scale bars represent 10 microns

Major cellular reprogramming breakthroughs now allow the reversal of terminally differentiated human cells (e.g. foreskin or skin fibroblasts, white blood cells) into pluripotency using reprogramming factors (Oct4, Sox2, Klf4, and c-myc) (Takahashi and Yamanaka 2006). Directed differentiation of human-induced pluripotent stem cells (iPSCs) into cardiomyocytes (iPSC-CMs) can be achieved by manipulation of Wnt signalling (Lian et al. 2012). iPSC-CMs can provide a long-term human in vitro model to investigate titin variants and their role in cardiomyopathy; they have the potential to mimic human pathophysiology as they are not affected by species-related physiological differences (such as heart rate and ion channel setup) between humans and rodents. However, foetal characteristics and immaturity are major drawbacks of iPSC-CMs, for review of this subject see Jiang et al. (2018), Yang et al. (2014). Tissue engineering techniques such as cardiac microtissue, engineered heart tissue and engineered human myocardium are 3D culturing techniques that enable more mature phenotypes of iPSC-CMs, e.g. in respect to sarcomere organisation (Giacomelli et al. 2017; Hansen et al. 2010; Tiburcy et al. 2017). Alternatively, nano-patterning on substrates of physiological stiffness (Ribeiro et al. 2015), scaffolds (Blazeski et al. 2019) or bioactive lipids (Sharma et al. 2018) have been shown to improve iPSC-CM maturation. However, iPSC-CM are unlikely to reach full characteristics of adult cardiomyocytes morphologically or physiologically (Fig. 2).

iPSC-CMs are particularly powerful when combined with genome-editing techniques such as Clustered regularly interspaced short palindromic repeat (CRISPR)/CRISPR-associated protein 9 (Cas9) or Transcription activator-like effector nucleases (Chen et al. 2015; Ding et al. 2013). In principle, there are two approaches to study the consequences of variants e.g. in titin: patient-derived iPSC-CMs carrying the disease-associated variant are compared to iPSC-CMs from healthy control individuals (ideally close, unaffected family members) and/or an iPSC line in which the disease-associated variant is reverted to the wildtype allele via genome-editing. An alternative approach is to introduce a disease-associated variant into iPSC derived from a normal individual and using isogenic un-edited or control-edited iPSC as controls. The advantage of the latter approach is that both hetero- and homozygous variants can be generated. Examples of both approaches will now be discussed:

iPSC-CMs derived from DCM patients carrying A-band TTNtv (Ser14450fsX and Ser19628IfsX) exhibited a marked reduction in contraction amplitude, alterations in calcium transients and sarcomeric structural disruption, with less iPSC-CM displaying fully organised sarcomeres compared to iPSC-CM from a healthy control individual (Gramlich et al. 2015; Schick et al. 2018). Rescue by skipping the affected exon with antisense oligonucleotides resulted in improved myofibril assembly and stability as well as in a normalised gene expression profile, similar to the functional rescue observed in a corresponding mouse model (see above) (Gramlich et al. 2015).

Hinson et al. (2015) carried out an investigation into the penetrance of two TTNtv (Ala22352fsX and Pro22582fsX) and one missense variant (Trp976Arg) in the A-band and Z/I-band junction of the sarcomere respectively using both patient-derived iPSC-CMs and genome-edited iPSC-CMs, both in 2D cultures and cardiac microtissue. Both TTNtv lines displayed significant reductions in contractile forces compared to wildtype iPSC-CMs although I-band TTNtv did not result in the same pronounced pathogenic phenotypes inferring a reduced penetrance (Hinson et al. 2015). A further study of an A-band TTNtv showed that the resultant shortened titin also displayed impaired contractile performance, poor sarcomere assembly and myosin mechanical force (Chopra et al. 2018).

Taken together, the small number of studies of DCM-associated titin variants in iPSC-CMs so far have revealed reduced contractility and impaired sarcomere assembly as contributors to the disease.

## New methodological developments: opportunities to advance the use of iPSC-CM for studying titin variants

Functional assessment of contractility is still an issue in iPSC-CM, as established methods used for adult mouse cardiomyocytes, which track changes of the visible striation of highly organised sarcomeres, fail in the less organised iPSC-CM. However, recent advancements of open-source contractility analysis software tools such as MuscleMotion and SarcTrack will help to shed light on the role of titin variants in cardiac contractility: MuscleMotion is an ImageJ plug-in allowing the label-free quantification of contraction of e.g. individual iPSC-CMs, 2D monolayers or engineered heart tissue based on analysing changes in pixel intensity relative to a reference frame (Sala et al. 2018). SarckTrack is a recent MatLab contractility software which allows hundreds of individual fluorescently-labelled sarcomeres in iPSC-CM to be analysed in each cell (Toepfer et al. 2019).

Moreover, recent diversifications of the CRISPR/Cas9 toolbox open new avenues to manipulate iPSC-CMs: its traditional application is in genome-editing, utilising non-homologous end joining or homologous directed repair (reviewed in Devkota (2018), Singh et al. (2017)). The former is commonly used to generate null alleles via small insertion/deletion disrupting the reading frame (Chopra et al. 2018), the latter is used to introduce fusion tags e.g. a green fluorescent protein titin fusion to visualise sarcomeres (Toepfer et al. 2019) or site-specific variants, e.g. TTNtv (Hinson et al. 2015).

The specificity of CRISPR is also captured as a site-specific molecular localiser. Different functional molecules can be tethered with catalytically deficient Cas9 (dCas9) and guided to the target locus without genetic perturbation (Knott and Doudna 2018; Larson et al. 2013). Firstly, the CRISPR activation system (CRISPRa) recruits positive transcription regulators to the promoter or proximal enhancer regions and thereby drives gene expression (Hilton et al. 2015; Konermann et al. 2015; Zhang et al. 2015). This technology wins over the conventional overexpression systems, which depend on a random integration of the expression vectors, as it does not affect the host’s DNA sequence. Various modified versions of this gene regulation tool have been developed to include different effectors, or in combination, to maximise the fold activation (Chavez et al. 2016; Hilton et al. 2015). Modelling disease systems with distinguished expression profiles and governing key transcription factor expression to direct differentiation (Weltner et al. 2018) are promising applications of CRISPRa.

On the contrary, CRISPR interference (CRISPRi), blocks transcriptional activity by a steric hindrance between dCas9 and the transcriptional machinery (Larson et al. 2013) or by the actions of tethered transcription repressors (Parsi et al. 2017). Compared to existing gene knockdown methods owing to various advantages: CRISPRi is more effective in gene silencing as it acts directly at the transcriptional status, while RNA interference modulates the expression at mRNA level (Agrawal et al. 2003). Moreover, RNA interference targets are limited to cytosolic mRNAs but CRISPRi holds a broader target repertoire, including coding and non-coding sequences (Gilbert et al. 2014). The modularity of CRISPR system makes it easy to create guide RNA libraries. Lastly, the repression activity can be fine-tuned by changing the target region or by probing multiple sites simultaneously—CRISPRi can even be switched on or off using inducible CRISPRi-KRAB (Parsi et al. 2017) if time-dependent study is needed for example to dissect developmental phases.

In summary, the CRISPR/Cas9 system provides exciting tools beyond conventional genome-editing to study e.g. the consequences of titin variants in the cardiac system.

## Conclusion

While a causal role for TTNtv in DCM is now well established, the underlying disease pathways are still not fully understood. Animal models have highlighted a crucial role of titin for the integrity of the heart and have helped to gain insight into aspects of titin’s function, such as sarcomere assembly and signalling and their perturbations by titin variants. However, animal models are limited by species-related differences in morphology and physiology to humans. As an alternative, studies of human cardiac tissue can also provide insights into the patho-mechanisms of titin variants, but such tissue is rarely available in sufficient quality and quantity. Therefore iPSC-CM emerge as a novel model system to study titin-related cardiomyopathy. These cells allow mimicking human genetic disease in a human cardiac cellular model. While still in its infancy and currently plagued by the issue of immaturity, it provides exciting approaches to study the role of titin variants in cardiomyopathies and we are looking forward to future developments in the field.

## Abbreviations

CRISPR: Clustered regularly interspaced short palindromic repeat
CRISPRa: CRISPR activation system
CRISPRi: CRISPR interference
DCM: Dilated cardiomyopathy
HCM: Hypertrophic cardiomyopathy
iPSC: Induced pluripotent stem cells
iPSC-CM: Induced pluripotent stem cell derived cardiomyocyte(s)
TTNtv: Titin truncating variant(s)
PSI: Percentage spliced in
RNAseq: RNA sequencing
